# Communicating Hydrocephalus as a Consequence of Chronic Kidney Disease on Maintenance Hemodialysis: An Uncommon Complication of a Common Disease

**DOI:** 10.7759/cureus.26187

**Published:** 2022-06-22

**Authors:** Siva Reddy, Anamika Giri, Nikhil Pantbalekundri, Sunil Kumar, Sourya Acharya

**Affiliations:** 1 Department of Medicine, Jawaharlal Nehru Medical College, Datta Meghe Institute of Medical Science (Deemed to be University), Wardha, IND

**Keywords:** cerebrospinal fluid, chronic kidney disease, ventriculoperitoneal shunt, haemodialysis, hydrocephalus

## Abstract

While many etiologies of hydrocephalus for different age groups have been studied in detail, chronic kidney disease remains rare. We report a case of a 42-year-old male who was a known case of chronic kidney disease on maintenance hemodialysis since seven years. He was brought to the emergency department with a history of altered sensorium and irrelevant talk since the last 4-5 hours and was found to be a case of communicating hydrocephalus with periventricular ooze, as revealed by a computed tomography (CT) of the brain. A ventriculoperitoneal shunt surgery was performed, and the patient ultimately recovered and was discharged.

## Introduction

Hydrocephalus is an abnormal swelling of the ventricles caused by a high amount of cerebrospinal fluid accumulating inside the skull. There is a wide range of etiologies behind the development of hydrocephalus, which include the obstruction in cerebrospinal fluid circulation known as obstructive hydrocephalus and decreased cerebrospinal fluid absorption known as communication hydrocephalus [1]. Normal pressure hydrocephalus is a disorder of chronic adult-onset communication hydrocephalus, which is formed mainly due to decrease in the cerebrospinal fluid absorption.

Chronic hydrocephalus in adults can manifest as of triad of gait disturbance, urinary incontinence, and dementia, which may or may not be associated with symptoms of increased intracranial signs such as headache and false localizing signs. Gait disturbance is the most common symptom of hydrocephalus [2].

In patients with end-stage renal illness, cognitive dysfunction is the most common complaint. End-stage renal disease (ESRD) has a two to three times increased prevalence of cognitive dysfunction than in the general population [3]. In patients with ESRD, hydrocephalus remains one of the main etiologies of dementia [2].

Only a few articles had reported the risk of hydrocephalus in patients with ESRD receiving long-term dialysis [3]. We report the case of a 42-year-old male who was known case of chronic kidney disease on maintenance hemodialysis having altered sensorium and irrelevant talk diagnosed as communicating hydrocephalus on CT scan of the brain.

## Case presentation

A 42-year-old male patient was brought to the casualty by his relatives with complaints of altered sensorium and irrelevant talk since the last 4-5 hours. The patient was a known case of chronic kidney disease and was on maintenance hemodialysis for the same since the last seven years, with the last dialysis done five days back. There was no history of any other comorbidities. There was also no history of fall or trauma to the head in the patient.

On examination, the patient was conscious but disoriented to time, place, and person. His blood pressure was 150/90 mmHg, heart rate was 56 beats per minute, normal jugular venous pressure was normal, and respiratory rate was 22 cycles per minute. There was no edema in the lower limbs. His pupils reacted appropriately to light and, there was no papilledema on fundus examination. There was no postural hypotension. Respiratory examination revealed bilateral crepts. Neurological examination showed mild cognitive function impairment with ataxic gait. The patient presented with slurred speech, and there was no evidence of any sensory deficit.

**Table 1:** Laboratory investigations

Parameters	Values
Hemoglobin	6.5 mg/dL
Mean corpuscular volume	87.8 fL
White blood cells	8,000 cmm
Platelets	1.27 lakh/cmm
Alanine transaminase	20 U/L
Aspartate aminotransferase	34 U/L
Total protein	7.3 gm/dL
Albumin	3.9 gm/dL
Alkaline phosphatase	73 U/L
Conjugated bilirubin	0.1 mg/dL
Total bilirubin	0.7 mg/dL
Urea	59 mg/dL
Creatinine	8.1 mg/dL
Sodium	135 mmol/L
Potassium	5 mmol/L
Cerebrospinal fluid glucose	20 mmol/L
Cerebrospinal fluid protein	302 gm/L
Cerebrospinal fluid lactate dehydrogenase	84 U/L
Cerebrospinal fluid pH	7.2

**Figure 1:**
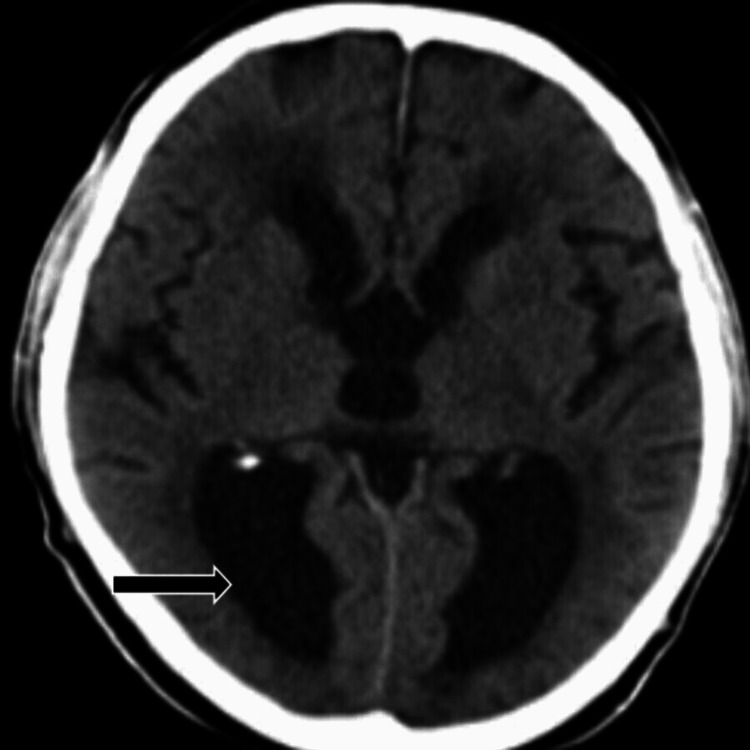
CT of the brain showing communicating hydrocephalus with periventricular ooze

CT of the brain was performed, which was suggestive of dilated bilateral lateral, third, and fourth ventricles, thus indicating communicating hydrocephalus with periventricular ooze (Figure 1).

The hydrocephalus was resolved, and the patient recovered well after a ventriculoperitoneal shunt was implanted. The prior antihypertensive medicines helped to keep blood pressure in check. During the perioperative period, nafamostat mesilate was used as an anticoagulant during dialysis, Three weeks later, the patient was switched back to heparin. He was discharged two weeks after admission in an improved state.

## Discussion

Congenital and acquired hydrocephalus, as well as communicating, non-communicating, and normal pressure hydrocephalus, are all different types of hydrocephalus [3,4]. It usually happens in the fifth or later decade of life. Hydrocephalus can be caused by a stroke, an injury, or a hemorrhage [2]. Communicating chronic hydrocephalus is a phenomenon that is currently poorly understood. The daily peak pressure is aberrant, most likely due to a lack of resorption. The resorption problem causes cerebrospinal fluid buildup without any increase in production. In healthy people, cerebrospinal fluid is produced predominantly by the choroid plexuses and secondarily by structures such as the ependyma. Several drainage routes are used to remove the cerebrospinal fluid. The recent discovery of barriers between the vascular and ventricular systems linked to extravasation from the vascular to the ventricular system is a possibility with active resorption surfaces, which could be an active or passive mechanism. Arachnoid granulations and the lymphatic system along the cranial nerves are part of the traditional resorption system, but current research is looking into the role of other resorption pathways in communicating chronic hydrocephalus, such as the glymphatic system along intracranial capillaries. It appears to play a role in the clearance of numerous compounds from the brain interstitium, including amyloid protein A42. Cerebrospinal fluid flow is lower in patients with communicating chronic hydrocephalus in the glymphatic system than in controls, and this decrease is linked to a decreased level of AQP4 expression, albeit the mechanism is unknown at this time. The perivascular regions appear to be affected by the decrease in AQP4. Changes in glymphatic drainage have been linked to the accumulation of compounds linked to Alzheimer's disease and communicating chronic hydrocephalus, while no link between the two disorders has been established. A hereditary form of communicative hydrocephalus has recently been identified, which is linked to a CFAP43 gene mutation. This mutation affects younger people, but further testing is needed to determine its role in this condition.

Hydrocephalus ex vacuo is a kind of hydrocephalus that occurs when the brain parenchyma shrinks and the ventricles dilate as a result of a stroke, trauma, or degenerative illness [3]. Because of his hypertension and heparin use during dialysis, our patient was at risk of intraventricular hemorrhage. Heparin administered during dialysis on day 3 could have led to intraventricular hemorrhage. Hydrocephalus can develop as a result of subarachnoid hemorrhage and meningitis, both of which are very common in ESRD patients and can produce inflammation and arachnoid granulation fibrosis, thus obstructing cerebrospinal fluid absorption [4]. A rare consequence of chronic kidney disease on hemodialysis can be normal pressure hydrocephalus, which presents with intensified headache, vomiting, or the typical neurologic presentation [5].

## Conclusions

Though a rare sequela of chronic kidney disease, hydrocephalus should be kept as an important differential in order to enable prompt diagnosis and management leading to prevention of mortality, such as in our case. Emergency ventriculoperitoneal shunt surgery may be advised for rapid recovery.
